# Gut inflammation in chronic fatigue syndrome

**DOI:** 10.1186/1743-7075-7-79

**Published:** 2010-10-12

**Authors:** Shaheen E Lakhan, Annette Kirchgessner

**Affiliations:** 1 Global Neuroscience Initiative Foundation, Los Angeles, CA, USA

## Abstract

Chronic fatigue syndrome (CFS) is a debilitating disease characterized by unexplained disabling fatigue and a combination of accompanying symptoms the pathology of which is incompletely understood. Many CFS patients complain of gut dysfunction. In fact, patients with CFS are more likely to report a previous diagnosis of irritable bowel syndrome (IBS), a common functional disorder of the gut, and experience IBS-related symptoms. Recently, evidence for interactions between the intestinal microbiota, mucosal barrier function, and the immune system have been shown to play a role in the disorder's pathogenesis.

Studies examining the microecology of the gastrointestinal (GI) tract have identified specific microorganisms whose presence appears related to disease; in CFS, a role for altered intestinal microbiota in the pathogenesis of the disease has recently been suggested. Mucosal barrier dysfunction promoting bacterial translocation has also been observed. Finally, an altered mucosal immune system has been associated with the disease. In this article, we discuss the interplay between these factors in CFS and how they could play a significant role in GI dysfunction by modulating the activity of the enteric nervous system, the intrinsic innervation of the gut.

If an altered intestinal microbiota, mucosal barrier dysfunction, and aberrant intestinal immunity contribute to the pathogenesis of CFS, therapeutic efforts to modify gut microbiota could be a means to modulate the development and/or progression of this disorder. For example, the administration of probiotics could alter the gut microbiota, improve mucosal barrier function, decrease pro-inflammatory cytokines, and have the potential to positively influence mood in patients where both emotional symptoms and inflammatory immune signals are elevated. Probiotics also have the potential to improve gut motility, which is dysfunctional in many CFS patients.

## Introduction

Chronic fatigue syndrome (CFS) is a clinical condition characterized by persistent and unexplained relapsing fatigue that is worsened by physical and mental exertion [1,2]. According to the Centers for Disease Control (CDC) report, the overall prevalence of CFS in the USA is as many as 4 million people [3,4]. Up to 80% of those affected are women [5]. These individuals suffer from severe fatigue that impairs daily activity, diminishes quality of life (QOL) for years, and has no known cure. CFS represents an economic burden for society and healthcare institutions [6].

The CDC originally proposed the term CFS in 1988. In 1994, the International CFS Study Group published a revised and more inclusive case definition which defines CFS on the fulfillment of two major criteria: chronic fatigue of at least 6 months duration, which is accompanied by various rheumatologic, infectious and neuropsychiatric symptoms [7]. Although considerable progress has been made in recent years, a major gap in the knowledge of the pathogenesis of CFS remains and has precluded the discovery of effective forms of treatment. Moreover, the diagnosis of CFS is highly problematic since no biological markers specific to this disease have been identified. Thus, CFS is a diagnosis of exclusion [7].

Along with disabling fatigue, CFS is characterized by neuropsychological disturbances such as headaches, myalgia, arthralgia, and post-exertional malaise; cognitive difficulties, with impaired memory and concentration; un-refreshing sleep; and mood changes, such as depression and anxiety [8]. In addition, many CFS patients complain of gastrointestinal (GI) disturbances, including abdominal pain or discomfort and an alteration in bowel habit. In fact, patients with CFS are more likely to report a previous diagnosis of irritable bowel syndrome (IBS), a common functional disorder of the gastrointestinal tract, and experience IBS-related symptoms [9]. Although the precise pathophysiology of CFS is yet to be fully elucidated, the high frequency of co-morbidities in CFS suggests that the underlying pathophysiological mechanisms cannot be confined to an organ (e.g., the brain), but rather must involve an integrating system or mechanism such as the brain-gut axis and the autonomic nervous system.

There is a growing body of literature describing immune dysfunction in CFS [10]. A possible involvement of the immune system is supported by the observation that the onset of CFS is often preceded by virus infections and a "flu-like" illness. For example, infectious mononucleosis has been shown to be a risk factor for developing CFS [11]. Immune activation is also suggested by an elevation of pro-inflammatory cytokines, an increased expression of T lymphocyte activation markers, such as CD26 and CD38 [12] and decreased function of natural killer (NK) cells (for review see [13]).

Both physical and psychological stressors have been found to activate the hypothalamic-pituitary-adrenal (HPA) axis, an important link between the brain and the immune system [14]. Corticotrophin-releasing hormone (CRH) produced in the hypothalamus, is the primary hypothalamic regulatory peptide of the HPA axis and its release has been shown to be controlled by circulating pro-inflammatory cytokines, especially interleukin (IL)-6 [15]. Thus, the increase in pro-inflammatory cytokines seen in CFS patients may be involved in an exaggerated activation of the HPA axis [14].

The frequent association between CFS and IBS could also be governed by elevated circulating pro-inflammatory cytokines acting either locally or on the brain-gut axis. Patients with IBS were shown to have increased plasma levels of IL-6 and IL-8 [16]. Moreover, patients with both IBS and CFS were found to have, in addition, increased levels of two other pro-inflammatory cytokines, IL-1β and tumor necrosis factor (TNF)-α [16]. Given that other studies have shown a variety of findings (mast cell activation, increased lymphocytes) suggesting a state of low-grade inflammation or immune activation in the mucosa and lamina propria of the bowel [17], increased serum concentrations of cytokines have been interpreted as evidence of spill-over from a primary focus in the gut. Thus, pro-inflammatory cytokines may be involved in the exaggerated activation of the HPA axis and thereby promote a coordinated central response to stress, such as GI inflammation and dysfunction.

There is now evidence that CFS is associated with marked alterations in the gut microbiota, with lower levels of *Bifidobacteria *and higher levels of aerobic bacteria [18]. Gut pathogens can communicate with the CNS and influence emotional behaviors such as anxiety and depression, even at extremely low levels and in the absence of an immune response [19,20]. In addition, probiotics or live microorganisms which confer a health benefit on the host, have the potential to decrease mood-regulating systemic pro-inflammatory cytokines, decrease oxidative stress and improve nutritional status when orally consumed (see below; [18]). Probiotic lactic acid producing-bacteria have been shown to prevent and alleviate GI disturbances and to normalize the cytokine profile which might be of an advantage for patients suffering from CFS [21].

In the case of CFS, evidence for a synergism between aberrant gut microbiota, mucosal barrier dysfunction, and altered mucosal immunity contributing to the disorder's pathogenesis has begun to evolve. Research shows that patients with CFS have alterations in gut microbiota [22]. Moreover, emerging studies have demonstrated that pathogenic and non-pathogenic gut microbiota may influence mood-related symptoms and even behavior in animals and humans. These findings lend further support to the presence of a gut-brain interface, one that may be modulated by gut microbiota.

Experimental data also show that inflammation, even if mild, can lead to persistent changes in GI nerve and smooth muscle function, resulting in dysmotility, hypersensitivity, and dysfunction. Furthermore, alterations in gut function are observed after the resolution of an acute intestinal inflammation [23,24]. The enteric nervous system (ENS), the intrinsic innervation of the bowel, controls virtually all GI functions (e.g., motility, secretion, blood flow, mucosal growth and aspects of the local immune system). Whether the persistent alterations in gut function observed in the majority of CFS patients are due to inflammation-related changes in the properties of enteric nerves is unknown.

This review will provide a brief overview of the current understanding of the role of gut inflammation in CFS. Despite advances in the understanding of the pathophysiology of CFS, therapeutic options for combating the symptoms of CFS are still not available.

## Viral infection

Early conceptualizations of CFS focused on the role of viral infection. This is not surprising since in 60 to 80% of published reports, CFS presents with acute onset of illness, with systemic symptoms similar to influenza infection that do not subside. Numerous researchers have tried to correlate infection by several microorganisms with the onset of CFS. For example, the human parvovirus (HPV)-B19 has been the most reported CFS-associated virus [25]. Although several studies have detected parvovirus B19 DNA in the GI tract of CFS patients, it is not consistently detected in all patients [25]. Another difficulty is associating the onset of CFS with the presence of antibodies to HPV-B19.

Other studies have suggested that infection by another virus, the human herpes virus-6 (HHV-6), a neurotropic, gliotropic, and immunotropic virus, is more often found in patients with CFS than in healthy controls [26]. However, using real-time PCR, high loads of HHV-6 DNA were detected in most CFS and control biopsies [27]. Other studies attempted to show an association between Epstein-Barr virus (EBV) infection and postinfectious onset of CFS. EBV infection has been shown to cause extreme fatigue during the acute illness and to be a risk factor for developing CFS, with a prevalence rate of 8% observed at 6 months [11,28]. However, EBV was found in 15-30% of all biopsies [25]. Thus, the involvement of EBV in addition to various enteroviruses, and the human T-lymphotropic virus type 2 (HTLV-2) has not been conclusively proven [29].

In October 2009, Lomardi et al. reported finding a gammaretrovirus in peripheral blood mononuclear cell (PBMC) DNA from about 67% of CFS patients compared to only 3.6% of healthy persons using PCR testing [30]. The agent was named xenotropic MLV-related virus (XMRV) because its *env *gene was nearly identical to that of xenotropic MLV, an infectious endogenous MLV that preferentially infects cells from foreign species, including humans. Almost half of the CFS patients in this study described the onset of their symptoms as related to an acute viral disease. In addition, virus isolation and antibody detection were reported in some CFS patients.

Confirmation of an association and etiologic role of XMRV in CFS is important because it could provide a useful diagnostic test and might lead to new treatment interventions. Inhibitors of XMRV such as the integrase inhibitor raltegravir, are now available. However, two recent studies from the United Kingdom using PCR testing alone or together with serologic testing reported negative XMRV results in CFS patients [31,32]. XMRV was also not found by PCR testing in CFS patients from the Netherlands [33], China [34], or the United States [35], questioning the association of XMRV with CFS.

## Endotoxemia

Although confidence in the link between infection and CFS pathogenesis has waned [8], recent studies have suggested that infection with gut pathogens could be related to CFS onset.

Raised serum concentrations of IgA and IgM to lipopolysaccharide (LPS) of gram-negative enterobacteria, such as *Pseudomonas aeruginosa*, *Morganella morganii, Proteus mirabilis, Pseduomonas putida, Citrobacter koseri, *and *Klebsiella pneumoniae *have been reported in CFS patients [12]. The prevalence and median values for serum IgA against the LPS of enterobacteria were significantly greater in patients with CFS than in normal volunteers and patients with partial CFS. Moreover, serum IgA levels were significantly correlated to the severity of illness, as measured by the FibroFatigue scale [12]. The FibroFatigue scale is an observer's rating scale with 12 items measuring pain, muscular tension, fatigue, concentration difficulties, failing memory, irritability, sadness, sleep disturbances, and autonomic disturbances and irritable bowel, headache, and subjective experience of infection [36]. It is a reliable and valid measuring instrument that is used to monitor symptom severity and change during treatment of CFS patients [36].

Normally, the intestinal epithelium acts as a continuous barrier to avoid LPS translocation; however, some endogenous or exogenous events may alter this protective function [37]. This may induce an increased bacterial translocation and thus increased serum endotoxin concentrations which, in turn may trigger an immune response [38]. Thus, the increased serum IgA and IgM levels against LPS in CFS indicate the presence of increased gut permeability and an immune response mounted against LPS of the enterobacteria [12].

## Gut microbiota

The human GI tract contains a complex and delicately balanced ecosystem of more than 17 bacterial families encompassing 400 to 500 different microbial species. The main genera of these commensal bacteria are: *Lactobacillus, Bifidobacteria, Bacteroides, Clostridia, Fusobacteria, Eubacteria, Peptococcus, Streptococcus, Escherichia *and *Veillonella*. They regulate a myriad of host processes and provide several nutrients to their host and their symbionts within the microbial community. In healthy individuals, these relationships are thought to occur in equilibrium; however, the normal balance of gut microbiota can be altered by a number of factors and this is turn can contribute to certain functional disorders [39]. For example, the number of different commensal bacteria is altered in inflammatory bowel disease (IBD). IBD patients have increased bacteroides, adherent or invasive *Escherichia coli*, and enterococci, and reduced *Bifidobacteria *and *Lactobacillus *species [40].

The composition of the gut microbiota can be altered by various factors including stress. Psychological stress alters the gut microbiota towards decreased numbers of *Bifidobacteria *and *Lactobacilli *[41]. *Bifidobacteria *are a group of bacteria that have been shown to reduce intestinal LPS levels and LPS-induced activation of nuclear factor-kappa B (NF-κB) in mice [42]. Inhibition of LPS-induced NF-κB activation was accompanied by a dose-dependent decrease of pro-inflammatory cytokines and cyclooxygenase 2 [42]. Stress in neonatal Rhesus monkeys was reported to suppress the numbers of *Lactobacilli *in the fecal flora in association with increased susceptibility for opportunistic infections [43]. Restraint conditions, acoustic stress and food deprivation have all been shown to negatively alter gut microbiota in various animal studies [44,45]. Interestingly, stress (e.g., psychological, physical exhaustion) is a well-established trigger factor for CFS [12].

Investigations have documented that there are marked alterations in the gut microbiota of CFS patients, with lower levels of *Bifidobacteria *and higher levels of aerobic bacteria [18]. Dr. Henry Butt and colleagues from the University of Newcastle, Australia have been examining the intestinal microbiota of CFS patients for a number of years. In 1998, they presented the first evidence of altered fecal microbiota in CFS patients compared to normal, healthy controls [46]. The mean distribution of the Gram negative *Escherichia coli *as a percentage of the total aerobic flora of control subjects was 92.3% compared to 49% in CFS patients. Among aerobes, the D-lactic acid producing *Enterococcus *and *Streptococcus *species were strongly over-represented in CFS patients. These findings were recently confirmed [22]. Among anaerobic bacteria, *Prevotella *was the most commonly overgrown bacteria. Moreover, it was shown that the higher the aerobic enterococcal count, the more severe the neurological and cognitive deficits including nervousness, memory loss, forgetfulness and confusion [46]. Consequently, high plasma LPS levels in CFS could result from an increased production of endotoxin upon changes in the gut microbiota [47].

## Mucosal barrier function

To protect itself from uncontrolled inflammatory responses, the intestinal epithelium has developed mechanisms to restrain bacterial growth, limit direct contact with the bacteria, and prevent bacterial dissemination into underlying tissue. The mucosal barrier, which consists of only a single layer of epithelial cells, is one of the most important components of the innate immune system, and all that separates the inside of the body from a very "dirty" outside environment. Thus, mucosal barrier function is a key component in the arsenal of defense mechanisms required to prevent infection and inflammation. Mucosal barrier function is maintained by several interrelated systems, including mucous secretion, chloride and water secretion, and binding together of epithelial cells at their apical junctions by tight junction proteins. Together, they act as the "gatekeeper" of the mucosal barrier.

Disruption of mucosal barrier function occurs in CFS as demonstrated by the increased serum concentrations of IgA and IgM to LPS of gram-negative enterobacteria [12]. Psychological stress disrupts the mucosal barrier allowing increased entry of antigens and microorganisms, which in turn is expected to stimulate hyperactive responses in the mucosal immune system. For example, chronic water avoidance stress in rats induces increases in the adherence of bacteria to intestinal epithelial cells, bacterial internalization into enterocytes and the appearance of bacteria in the lamina propria [48]. Consequently, mucosal barrier dysfunction causes alterations in gut motility, abnormal secretion, and changes in visceral sensation that could contribute to symptom generation. This may at least partially explain the link between stress and CFS. The relationship between IBS and CFS may also reflect in part disorders in gut permeability as altered gut microbiota (e.g., higher numbers of *Veillonella *and *Lactobacillus *than healthy controls) and a disrupted mucosal barrier are found in patients with IBS [49,50]. Furthermore, IBS patients with high acetic acid or propionic acid levels presented more severe symptoms, impaired quality of life and negative emotions [50].

Butt and colleagues reported that fatigue presentation in CFS patients with symptoms of IBS was more severe than in CFS patients without irritable bowel [46]. Furthermore, patients with both CFS and IBS had poorer appetite, increased abdominal pain, increased severity of loose stools, diarrhea, nausea, and gastric reflux. The gut microbiota influences the sensory, motor and immune system of the gut and interacts with higher brain centers even at extremely low levels [50]. So aberrant gut microbiota and gut barrier dysfunction may actually be creating an "irritable" bowel. Altered intestinal microbiota and gut barrier dysfunction could also contribute to the symptoms of CFS through increased translocation of LPS from gram-negative enterobacteria.

## Therapeutic restoration of mucosal barrier function

Since altered intestinal microbiota and gut barrier dysfunction barrier are found in CFS [18], they offer potential targets for intervention that would include modulation of the gut microbiota to correct an imbalance, as well as tightening of interepithelial junctions. Enhancement of barrier function by probiotic bacteria has been observed in both *in vitro *models and *in vivo *animal models [51].

Probiotics are live microorganisms with a vast array of therapeutic potential for GI disease. They have a beneficial effect on the intestinal mucosa via several proposed mechanisms that include inhibition of the mucosal adhesion of pathogens, improvement of the barrier function of the epithelium, and alteration of the immune activity of the host. They may also regulate intraluminal fermentation and stabilize the gut microbiota [39]. In addition, probiotics have recently emerged as promising adjunctive therapy in treating IBS, with *B. infantis *becoming the frontrunner for treatment (for review see [52]).

Probiotic bacteria are *Lactobacilli spp*., certain types of *Streptococcus*, and *Bifidobacteria spp*., but also other non-pathogenic bacilli such as *E. coli*-Nisle 1917 and yeasts such as *Saccharamyces boulardii*. They secrete short chain fatty acids, an action that results in decreased luminal pH and production of bactericidal proteins. Butyric acid, a byproduct of bacterial fermentation of fiber, has been shown to nourish colonic enterocytes, enhancing mucosal integrity [53]. In addition, probiotics may improve bowel dysmotility [53].

Researchers have demonstrated the utility of probiotics for mood regulation in CFS patients [54]. Administration of *Lactobacillus casei *strain Shirota (LcS; 24 billion cfu/day) to adult patients meeting the formal diagnostic criteria for CFS, was found at eight weeks to cause a significant rise in both *Lactobacillus *and *Bifidobacteria *in those taking the LcS and there was also a significant decrease in anxiety symptoms [54]. The elevation of *Bifidobacteria *levels should be considered a positive finding, particularly when considering that *Bifidobacteria *levels may be low in CFS [18].

*Bifidobacteria *appear to play an important role in maintaining the gut barrier. An increase in *Bifidobacteria *in *ob/ob *mice was associated with a significant improvement of gut permeability measured *in vivo*; this improvement was linked to an increase in tight junction mRNA expression and protein distribution [47]. In addition, the rise in *Bifidobacteria *was correlated with a decrease in plasma LPS concentrations; therefore, a significant reduction in markers of oxidative and inflammatory stress [47].

*Bifidobacterium infantis *can boost serotonin levels in areas of the brain associated with anxiety and depression. Improvements in anxiety scores among those CFS patients consuming LcS bacteria are especially noteworthy [54]. The idea that implanting the gut with *Lactobacillus *strains may improve quality of life and mental health is not a new one. Dr. George Porter Phillips first reported in 1910 that although *Lactobacillus *tablets and powder were ineffective, a gelatin-whey formula with live lactic acid-producing bacteria improved depressive symptoms in adults with melancholia [55]. Overall, probiotics will likely have an emerging therapeutic role in treating CFS.

## Cytokines and inflammation

CFS is typically characterized by a chronic, low-grade inflammation. Although fatigue severity appears to correlate with inflammatory disease activity and is therefore consistent with an immunological model of CFS [12], it is not known whether inflammation causes fatigue. Results from a population-based study indicate that people with CFS had increased markers of peripheral inflammation when compared to healthy controls, but had a similar inflammatory profile when compared to unhealthy subjects who did not meet the criteria for CFS [56].

It is unlikely that CFS "causes" increased inflammation. Rather people with fatiguing conditions are likely to exhibit "unwellness" symptoms for a variety of reasons. One such reason may be an increase in peripheral pro-inflammatory signaling based on overwhelming evidence that pro-inflammatory cytokines are capable of inducing all the cardinal symptoms of CFS in humans [57,58]. Thus, factors that increase inflammation, such as stress and depression can increase peripheral inflammation, significantly increasing the risk in individuals with CFS of subsequently developing vascular disease, metabolic disease and dementia.

Several groups have reported evidence of chronic immune dysfunction in CFS although the exact nature of this dysfunction remains unclear. While initial studies generally suggested immunosuppression [59], recent years have seen increased interest in the possibility that activation of the innate immune response might contribute to symptom development in CFS [59,60]. Studies have reported increased plasma concentration of the acute phase reactant c-reactive protein (CRP) in patients with CFS. Other findings include higher frequencies of various autoantibodies. A significant increase in the numbers of B cells with CD20+ CD5+ phenotype which is correlated with autoantibody production and with CD21 markers that is a ligand for retroviruses was also found in patients with CFS [61].

CFS has a propensity to over-produce pro-inflammatory cytokines (e.g., TNF-α coupled with a decreased production of anti-inflammatory cytokines. A principal avenue of investigation has been the measurement in blood of immune signals conducted by cytokines. In animal studies, administration of pro-inflammatory cytokines (IL-1, TNF-α, and IL-6) directly into the brain can induce "sickness behaviors" that strongly resemble the symptoms of CFS. In particular, decreased motor activity, altered food and water intake, sleep and cognition have been linked to increases in the levels of IL-1b, IL-6 and TNF-α [57]. Furthermore, in humans, systemically administered pro-inflammatory cytokines, such as IL-6 and TNF-α typically induce a systemic inflammatory response where one of the major symptoms is intense fatigue.

The sensation of fatigue or exhaustion is prevalent in a number of infectious and chronic inflammatory disease states including IBD, systemic lupus erythematosus and ankylosing spondylitis [62]. In rheumatoid arthritis and IBD, in which TNF-α plays an integral role, fatigue is markedly improved by treatment with anti-TNF-α agents. Thus, amelioration of fatigue is mediated through an anti-inflammatory effect showing that the CNS is extremely susceptible to immunological reactions that occur during disease and injury.

The TNF-α inhibitors are a group of drugs that may provide benefit in CFS. One TNF-α inhibitor, etanercept, has been used with considerable benefit in the treatment of six patients with CFS in a pilot study [63]. Unfortunately, this trial was not published as a paper but only as a meeting abstract. The use of TNF-a inhibitors in CFS is strongly supported by data on the immune responses in CFS, and data from gene expression studies [64,65]. Thus, it is important to repeat this work and carry out a larger clinical trial of etanercept in patients with CFS.

On the basis of the results of gene expression studies, a clinical trial of IFN-β was proposed in CFS patients. IFN-β is associated with the regulation of humoral immune responses and immune responses against viral infections. IFN-β stimulates the activity of NK cells, which are considered to be inefficient in patients with CFS. It selectively inhibits the expression of some mitochondrial genes that are implicated by gene studies in patients with CFS [65]. Finally, IFN-β is a licensed treatment for multiple sclerosis, helping in reduction of fatigue. The pathogenesis of multiple sclerosis is also thought to be cytokine mediated, as has been shown in CFS [65].

Broderick et al. recently demonstrated that CFS is associated with a profound imbalance in the regulation of immune function [66]. In this study, instead of analyzing immunological markers individually, network analysis was applied to study the co-expression of 16 cytokines in CFS subjects and healthy controls. Analysis showed consistent and significantly attenuated patterns of Th1 and Th17 immune responses in CFS in the context of a well-established Th2 inflammatory milieu. These patterns would have escaped detection had the analysis focused solely on differential expression of individual cytokines.

Interestingly, the cytokine co-expression patterns described in this study, were consistent with the disruptive effects of latent viral infection by pathogens such as EBV. Viral triggers such as EBV have long been suspected of involvement in the onset and persistence of CFS. However, this virus is not consistently detected in all patients [25]. While other causes may underlie the cytokine expression pattern observed in CFS patients many of these are at least consistent with some of the disruptive effects of chronic viral infection.

## Effects of inflammation on the gut

Patients with CFS manifest symptoms suggestive of disturbed gut function, such as abdominal pain, diarrhea and/or constipation [9]. Under both physiological and pathological conditions, the ENS, the intrinsic innervation of the bowel, regulates intestinal mucosal function and coordinates the activity of the GI tract. The ENS is a component of the autonomic nervous system with the unique ability to function independently from the CNS (for review, see [67]). Enteric ganglia are organized into two major ganglionated plexuses, namely the myenteric (Auerbach's) and submucosal (Meissner's) plexus, and contain a variety of functionally distinct neurons, including primary afferent neurons, interneurons, and motor neurons, synaptically linked to each other in microcircuits.

While the myenteric plexus mainly regulates intestinal motility, the submucosal plexus together with nerve fibers in the lamina propria are involved in regulating epithelial transport. These nerves form networks within the lamina propria of both crypts and villi with the terminal axons in close contact with the basal lamina, an ideal position not only to affect epithelial cell functions but also to detect absorbed nutrients and antigens. These substances or released mediators from epithelial cells may act on the nerve terminals to change the properties of enteric neurons and cause peripheral sensitization. Consequently, permanent or even transient structural alterations in the ENS disrupt normal GI function. Since the ENS controls the motility and secretion of the bowel these abnormalities indicate the impact of inflammation on neural signaling in the ENS.

Several studies have demonstrated ENS structural changes associated with gut inflammation. For example, damage to axons has been observed in the inflamed human intestine in episodes of IBD [68-70]. Other changes that occur in the ENS during inflammation include altered neurotransmitter synthesis, content, and release, changes in glial cell numbers and a myenteric ganglionitis associated with infiltrates of lymphocytes, plasma cells and mast cells [71,72]. In fact, consequences of intestinal inflammation, even if mild, persist for weeks beyond the point at which detectable inflammation has subsided (for review see [73]). Thus, persistent changes in GI nerve function, resulting in dysmotility, pain, and gut dysfunction long after the resolution of the initiating inflammatory event could contribute to GI disorders observed in CFS.

## Oxidative stress

It is well known that CFS is accompanied by increased oxidative stress. People with chronic conditions, such as CFS have lower levels of L-glutathione [74]. Research has shown that L-glutathione levels in cells can be dramatically depleted by excessive oxidative stress. In turn, L-glutathione deficiency has been shown to contribute to oxidative stress and disease, resulting in a vicious cycle.

Oxidative stress arises when there is a marked imbalance between the production of reactive oxygen species (ROS) and their removal by antioxidants. In reaction to mild oxidative stress, tissues often respond by producing more antioxidants; however, severe persistent oxidative stress depletes body antioxidant resources and overtakes its ability to produce more antioxidants, leading to lower antioxidant levels and as well as injury in the tissues.

8-OH-deoxy guanosine (8-OHdG) is a commonly used and highly sensitive marker of total oxidative stress in the body. Upon DNA repair, 8-OHdG is excreted in the urine. Numerous studies have indicated that urinary 8-OHdG is not only a biomarker of generalized cellular oxidative stress, but also a risk factor for many diseases including CFS. Elevated urinary 8-OHdG DNA was detected in patients with CFS. Moreover, the level of urinary 8-OHdG in CFS correlated with the severity of depression and malaise [75]. Thus, increased activation of oxidative and nitrosative (IO and NS) pathways, plays a role in CFS. Moreover, measuring urinary 8-OHdG may be a convenient method for evaluating oxidative DNA damage in patients with CFS and could be a sensitive biomarker helpful for the early diagnosis of patients with CFS.

Maes proposed that IO and NS pathways play a key role in the pathophysiology of CFS [76]. Increased plasma concentrations of pro-inflammatory cytokines, oxidative damage, increased COX-2 production, and increased translocation by gram-negative enterobacteria can generate CFS-like symptoms including fatigue, a flu-like malaise, pain, symptoms of IBS, and neurocognitive disorders. In addition, aberrations in IO and NS pathways are interrelated.

For example, viral and bacterial infections and gut-derived inflammation may induce NF-κB and consequently COX-2, inducible nitric oxide synthase (iNOS) and increased levels of pro-inflammatory cytokines. These inflammatory pathways and persistent or reactivating infections induce ROS and radical nitrogen species (RNS), which in turn may damage membrane fatty acids, proteins, DNA and mitochondria. As a consequence, some cellular immune functions may be suppressed, for example, lowered NK cell activity and *ex vivo *expression of T cell activation markers, such as CD69. Depletion of antioxidants in patients with CFS, partially due to inflammation, may further impair the protection again ROS and RNS, causing more damage to fatty acids, proteins, DNA and mitochondria [76].

## Hydrogen sulfide

Alterations of the gut microbiota may have serious consequences for the host health. Overgrown pathogenic bacteria found in the oral cavity and GI tract produce the "toxic gas" hydrogen sulfide (H_2_S) when they come in contact with heavy metals. H_2_S, a colorless, flammable and water-soluble gas with the characteristic odor of rotten eggs, has been known for decades because of its toxicity and as an environmental hazard [77]. Inhibition of mitochondrial respiration, more potent than that of cyanide, resulting from blockade of cytochrome c oxidase is the main mechanism of H_2_S toxicity [78].

H_2_S is normally found in the body, which suggests that this molecule could have physiologic relevance. The mucosa of the gut is continuously exposed to H_2_S generated by sulfate-reducing bacteria [79]. However, too much H_2_S, produced by the overgrowth of harmful, pathogenic bacteria as occurs during inflammation causes the intestinal epithelial barrier to break down. Increased levels of bacterial H_2_S stimulate the production of destructive compounds called ROS, which inhibit mitochondrial function directly. An increase in ROS caused by an imbalance between antioxidant defenses and ROS production results in tissue damage and, eventually, cell death. This is a key mechanism for the development of gut infections. Thus, there is evidence that H_2_S is involved in chronic (long-term) inflammation of the gut [80].

People with CFS were shown to have higher concentrations of intestinal bacteria than normal, which probably leads to higher levels of H_2_S. Professor Kenny De Meirleir of the Brussels Free University and his team say high levels of H_2_S caused by an intestinal overgrowth of Gram positive D/L lactate-producing bacteria play a major role in CFS and lead to a series of reactions in your body that leave cells devoid of oxygen and energy.

Understanding the role of the intestinal barrier and its breakdown is an area of research that is currently receiving a great deal of attention as is considered by many scientists as the real basis for CFS [12]. Interestingly, it has recently been shown that H_2_S is also present in gut nerves in humans and guinea pigs. Schemann and colleagues found that more than 90% of neurons in the ENS contain enzymes that produce H_2_S [81]. In addition, H_2_S increased neuronal electrical activity and significantly increased mucosal secretion. These finding suggest H_2_S is a novel gut-signaling molecule and may be the third gaseous transmitter in addition to nitric oxide and carbon monoxide.

## Mitochondrial failure and CFS

Dr. Sarah Myhill and colleagues have proposed that CFS is linked to "mitochondria failure" [82]. Mitochondria use fuel molecules derived from food to produce energy by oxidative metabolism in the form of adenosine triphosphate or ATP, which when hydrolysed to the disphosphate, ADP, releases energy to produce muscle contractions, nerve impulses and all the energy-consuming processes needed to synthesize all of the complex molecules of the body. ATP recycles approximately every 10 seconds in a healthy individual. However, when mitochondria are impaired the energy they supply will be impaired and so the individually has poor stamina, as in CFS. Also, when a cell is filled with defective mitochondria, it not only becomes deprived of energy (ATP). It can accumulate a backlog of unused fuel molecules (glucose) and oxygen with disastrous effects. If the body is short of ATP, it can make a very small amount directly from glucose by converting it into lactic acid. This is exactly what many individuals with CFS do. They readily switch into anaerobic metabolism [83]. However, this results in serious problems.

The buildup of lactic acid in the blood, called lactic acidosis is associated with muscle pain, heaviness, aching and soreness ("lactic acid burn"), and might actually damage muscle and nerve tissue. People suffering from CFS have difficulty increasing their fitness [84]. Their response to incremental exercise is increased oxidative stress with marked alterations of muscle membrane excitability. Thus, mitochondrial dysfunction results in fatigue and could produce symptoms of CFS.

There is considerable evidence that mitochondrial dysfunction is present in some CFS patients. Muscle biopsies have shown abnormal mitochondrial degeneration in CFS patients [82]. Mitochondrial dysfunction occurs in neutrophils in CFS patients [82]. Neutrophils are the major effector cells of the immune system and the observed mitochondrial dysfunction is bound to have a deleterious effect on this system. Moreover, the degree of dysfunction was strongly correlated with the severity of their illness suggesting that the severity of a person's CFS relates to the severity of the mitochondrial dysfunction [82].

## Coenzyme Q10

Deficiency of coenzyme Q10 (CoQ10) has been found in some CFS patients [85]. CoQ10 is a mitochondrial nutrient, which acts as an essential cofactor for the production of ATP and displays significant antioxidant activities and anti-inflammatory effects.

In a study conducted by Maes and colleagues, more than 40% of patients with CFS had values below the lowest CoQ10 value detected in normal, healthy controls (490 microg/L). Moreover, CFS patients with very low plasma CoQ10 (< 390 microg/L) suffered significantly more from concentration and memory disturbances [85]. Deficiency of CoQ10 is also found in fibromyalgia, depression and a risk factor for atherosclerosis [86]. The presence of CoQ10 deficiency may also explain the accompanying depression seen in CFS and increased morbidity due to chronic heart failure in CFS patients. These results suggest that people with CFS might benefit from CoQ10 supplementation in order to normalize the low CoQ10 syndrome.

CoQ10 supplementation also appears to have a beneficial effect on gut dysfunction observed in animal models of colitis [87]. Over 12 weeks, the mice experienced a recovery in loose stools and bleeding in the GI tract. Since CoQ10 acts as an antioxidant, it may help lessen the inflammation by reducing tissue damage caused by gut pathogens. Future studies are required to determine whether oral CoQ10 is beneficial in the prevention and/or treatment of symptoms associated with CFS.

## Conclusion

Clearly, much work remains in terms of understanding the role of gut inflammation in CFS development. Despite several studies showing health benefits of probiotics, the enthusiasm for their application as well as other means to alter the intestinal microbial ecosystem in the prevention of CFS needs to be tempered due to the current lack of knowledge of the normal gut microbiota, in addition to questions as to how it affects the immune system. Future studies using newly developed techniques to evaluate the gut microbiota in CFS patients and/or animal models of CFS are certainly needed. In addition, research on the effects of mucosal barrier dysfunction on enteric nerves and their activity in CFS are clearly warranted.

## Abbreviations

CDC: Centers for Disease Control; CFS: chronic fatigue syndrome; CoQ10: coenzyme Q10; CRH: corticotrophin-release hormone; COX-2: cyclooxygenase-2; ENS: enteric nervous system; EBV: Epstien-Barr virus; GI: gastrointestinal; GSH: glutathione hypothalamic-pituitary-adrenal; HPA: human parvovirus B19; HPV: human herpes virus-6; HHV-6: human T-lymphotropic virus type 2; HTLV-2: hydrogen sulfide; H_2_S: inducible nitric oxide synthase; iNOS: inflammatory bowel disease; IBD: irritable bowel syndrome; IBS: interleukin-6; IL-6: interleukin 8; IL-8: interleukin1-beta; IL1-β: interferon-gamma; IFN-γ: interferon-beta; IFN-β: lipopolysaccarhide; LPS: natural killer; NK: nuclear factor-kappa B; NF-κB: peripheral blood mononuclear cell; PBMC: reactive oxygen species; ROS: tissue transglutaminase; tTG: tumor necrosis factor alpha; TNF-α: xenotropic MLV-related virus; XMRV: 8-OH-deoxy guanosine, 8-OHdG.

## Competing interests

The authors declare that they have no competing interests.

## Authors' contributions

All authors participated in the preparation of the manuscript, and read and approved the final manuscript.
